# *In vitro* simulation of the bioavailability of fluoride in food roasted with high-fluoride fuel and its health risks

**DOI:** 10.3389/fnut.2025.1560015

**Published:** 2025-04-09

**Authors:** Jianghui Zhang, Guomei Luo, Chenglong Tu, Na Yang, Kuo Tang, Chenghua Tu

**Affiliations:** ^1^School of Public Health (Key Laboratory of Environmental Pollution Monitoring and Disease Control, Ministry of Education), Guizhou Medical University, Guiyang, China; ^2^The Key Laboratory of Environment and Health, Guizhou Medical University, Guiyang, China; ^3^The Affiliated Hospital of Guizhou Medical University, Guiyang, China

**Keywords:** fluoride, food, bioavailability, health risk assessment, coal roasting

## Abstract

Fluorosis in coal-burning areas of Southwest China is exacerbated by multi-pathway fluoride exposure, with diet emerging as a critical factor due to traditional food preparation methods. This study systematically evaluated fluoride accumulation, bioavailability, and health risks associated with foods roasted over high-fluoride coal, with a particular focus on chili—a dietary staple with heightened fluoride absorption and bioavailability. Results indicated that after 30 days of roasting, chili's fluoride content reached 869.82 mg·kg^−1^, with bioavailability levels between 2.18 and 12.00%, making it the largest contributor to the hazard index (HI), accounting for over 80% of the total when bioavailability was considered. In comparison, corn, tofu, and pork, though accumulating fluoride to varying extents, presented lower bioavailability, and thus relatively lower health risks. Recent dietary shifts in the region show reduced corn consumption and increased chili and pork intake, further shifting dietary fluoride exposure risk toward high-bioavailability foods like chili. These findings highlight the need for dietary management interventions in high-fluoride regions, prioritizing moderated chili consumption and revised cooking practices to mitigate fluorosis risk effectively.

## 1 Introduction

Fluorosis is a widely recognized public health issue worldwide, particularly concerning in high-fluoride environments where multiple exposure pathways pose severe risks to skeletal and dental health (1–4). However, the causes and modes of fluoride exposure vary significantly across regions. In areas where high-fluoride coal briquettes, a composite fuel made from clay and coal, are commonly used, such as Southwest China, residents depend on these fluoride-rich briquettes for daily cooking and heating (5, 6). This briquette use results in substantial fluoride emissions, which enter the human body through multiple pathways, including air, drinking water, and diet, thereby leading to high rates of fluorosis, including chronic conditions like skeletal fluorosis and dental fluorosis (7). Compared to other forms of fluorosis, coal-burning fluorosis involves a complex mix of exposure sources, making it crucial to identify the primary exposure pathways to guide targeted interventions (5).

Despite the well-established understanding that coal-burning fluorosis results from multi-pathway exposure, significant debate persists regarding which route plays the most critical role in fluoride intake and associated health risks. The contributions of different exposure sources, including air, water, and food, to total fluoride intake among affected populations, are highly variable, which poses a challenge for precise health risk assessments (8–10). In a recent analysis, Yang et al. (11) examined fluoride exposure in Southwest China and identified air, drinking water, and diet as primary contributors to fluorosis, with food contamination during cooking emerging as a potentially pivotal pathway. Their findings suggest that traditional dietary practices, particularly those involving open-flame cooking with fluoride-rich coal, may be key factors in dietary fluoride exposure. This underscores the urgent need for accurate dietary exposure assessments that account for these traditional practices (11).

Fluoride ingestion through food leads to various biochemical and pathological changes, with the potential for long-term health impacts (12). Traditionally, health risk assessments of fluoride in food have been based on total fluoride content, yet this approach overlooks the actual absorption and bioavailability of fluoride within the body (13). Recent studies increasingly indicate that factors such as food matrix, cooking methods, and individual physiological status can significantly affect the absorption efficiency and metabolic processing of dietary fluoride, driving a need for bioavailability-based risk assessments (1, 14). Bioavailability, defined as the extent to which fluoride in food is absorbed and participates in biological activity within the body, offers a more precise estimate of potential health hazards than total content alone (15).

To enhance the accuracy of dietary fluoride risk assessments, recent studies have adopted the Caco-2 cell model to simulate intestinal fluoride absorption (16). This model reflects the characteristics of typical absorptive intestinal cells, providing a scientific basis for simulating fluoride absorption rates in the human gut. It serves as an effective tool for assessing the intestinal absorption efficiency and bioavailability of fluoride (17, 18). In this study, the Caco-2 cell model was used to simulate the kinetics of fluoride absorption from food in the human intestinal environment, providing insights into the actual absorption of fluoride during cooking and supporting the health risk assessment of dietary fluoride exposure.

To systematically analyze the dietary exposure risks for coal-burning fluorosis populations in Southwest China, a series of experiments were designed to explore the fluoride accumulation effects of major dietary components under high-fluoride fuel roasting conditions. Common local foods, including corn, tofu, pork, and chili, were selected to simulate fluoride deposition during typical cooking processes. The Caco-2 cell model was then used to quantify the bioavailability of fluoride from these foods, providing data on the intestinal absorption rates of fluoride. Additionally, health risk assessments incorporating point estimates and Monte Carlo simulations were conducted to quantify the potential health impact of dietary fluoride exposure. By integrating bioavailability into traditional fluoride exposure models and combining *in vitro* and probabilistic approaches, this study offers a novel perspective on the health risks of dietary fluoride exposure and informs public health strategies in regions affected by coal-burning fluorosis.

## 2 Materials and methods

### 2.1 Sample preparation and measurement

In June 2023, a total of 84 randomly purchased dietary samples of tofu, corn, pork, and chili peppers, with 21 samples of each, were collected in areas of typical fluorosis in Guizhou province, representing major local food sources. The samples were subjected to open roasting simulations using locally sourced high-fluoride briquettes (fluoride content: 850.23 ± 105.69 mg·kg^−1^). Fluoride content measurements were taken on days 0 (control), 5, 10, 15, 20, 25, and 30 (19). After sample collection, inedible parts were removed, and the samples were rinsed with ultrapure water, dried, and stored in sealed containers.

The fluoride content in samples was determined using the fluoride ion-selective electrode method as outlined in the Standard for Determination of Fluoride in Food (GBT 5009.18-2003) (20). The detection limit for this method was 0.01 mg·kg^−1^, with an average recovery rate of 104% and a relative standard deviation (RSD) of 2.1%.

### 2.2 Bioavailability

#### 2.2.1 UBM in vitro digestion

The European standardized method (unified BARGE method, UBM) is a standardized *in vitro* method based on the RIVM (the Dutch National Institute for Public Health and the Environment method) model, which was proposed in 2009 through a series of experiments. The steps are as follows (21, 22):

Gastric phase: Precisely weigh (0.1000 ± 0.0001) g of coal-roasted food sample and place it in a 50 mL centrifuge tube. Add 1.5 mL of simulated oral fluid (0.896 g KCl, 0.2 g KSCN, 0.888 g NaHPO4, 0.57 g Na_2_SO4, 0.298 g NaCl, 0.2 g urea, 145 mg α-amylase, 15 mg uric acid, 50 mg mucin, 0.95 mL NaOH (1.0 M), pH 6.5). After 5 min, add 2.25 mL of simulated gastric fluid (2.752 g NaCl, 0.266 g NaHPO4, 0.824 g KCl, 0.4 g CaCl_2_·2H_2_O, 0.306 g NH4Cl, 0.65 g glucose, 0.02 g glucuronic acid, 0.085 g urea, 0.33 g glucosamine hydrochloride, 1 g BSA, 1 g pepsin, 3 g mucin, 11.2 mL HCl (37%), pH 2.0). Mix thoroughly, then place in a shaker at 37°C and 100 rpm for 1 h.

Intestinal phase: After 1 h of gastric digestion, add 4.5 mL of simulated duodenal fluid (7.012 g NaCl, 5.607 g NaHCO3, 0.08 g KH_2_PO4, 0.564 g KCl, 0.05 g MgCl_2_, 0.1 g urea, 0.2 g CaCl_2_·2H_2_O, 1 g BSA, 3 g trypsin, 0.5 g lipase, 0.67 mL HCl (37%), pH 7.5), and 1.5 mL of simulated bile fluid (5.259 g NaCl, 5.785 g NaHCO3, 0.376 g KCl, 0.25 g urea, 0.222 g CaCl_2_·2H_2_O, 1.8 g BSA, 6 g bile salts, 0.18 mL HCl (37%), pH 6.5). Mix thoroughly and continue shaking at 37°C and 100 rpm for 4 h.

#### 2.2.2 Establishment of the Caco-2 cell model

Caco-2 cells were maintained in high-glucose DMEM medium supplemented with 20% fetal bovine serum, incubated at 37°C in a 5% CO_2_ atmosphere. The medium was refreshed every 48 h. Digestive fluids from chili, corn, white tofu, dried tofu, and pork were mixed with HBSS buffer at the following ratios: 0:1, 4:1, 2:1, 1:1, 1:2, 1:4, 1:5, and 1:0. These dilutions were applied to the cells, and cell viability under different dilution treatments was assessed using the CCK-8 assay.

In a 12-well transwell plate, 0.5 mL of complete medium was added to the upper chamber and 1.5 mL to the lower chamber, then incubated overnight at 37°C in a 5% CO_2_ incubator. Logarithmic-phase cells were diluted to a concentration of 1.25 × 105 cells/mL. 0.5 mL of the cell suspension was added to the upper chamber of the transwell insert, while 1.5 mL of complete medium was added to the lower chamber. The cells were incubated at 37°C in a 5% CO_2_ atmosphere with the medium changed every 48 h. Transepithelial electrical resistance (TEER) values were measured on 3, 5, 7, 9, 11, 13, 15, 17, 19, and 21 days during the establishment of the Caco-2 monolayer model (23).

#### 2.2.3 Bioavailability

The cell surface was rinsed 2–3 times with pre-warmed HBSS solution at 37°C and then equilibrated for 15 min. Following equilibration, 0.5 mL of the intestinal digestive fluid and HBSS mixture was added to the upper chamber of the transwell, and 1.5 mL of HBSS buffer was added to the lower chamber. After a 2 h incubation, the solution on the AP side was collected, and the fluoride content was measured using a fluoride ion-selective electrode, according to Equation 1 (24):


(1) $$\begin{array}{l} \text{RBA}\left(\text{\%}\right)=\frac{\left({\text{C}}_{\text{IV}}-{\text{C}}_{\text{AV}}\right)\;\times \;{\text{V}}_{\text{IV}}}{{\text{T}}_{\text{S}}\;\times \;{\text{M}}_{\text{S}}}\;\times \;100\text{\%} \end{array}$$

where *C*_IV_ is the concentration of fluoride in the intestinal digestive fluid (mg·L^−2^), *C*_AV_ is the fluoride concentration in the AP side solution during the bioavailability experiment (mg·L^−2^), *C*_AV_ is the volume of intestinal digestive fluid added (L), T_S_ is the fluoride concentration in the sample (mg·kg^−1^), and T_S_ is the mass of the sample added to the reaction system (kg).

### 2.3 Health risk assessment

#### 2.3.1 Points estimate of non-carcinogenic risk

In this study, points estimate of health risks was conducted to evaluate the non-carcinogenic risk (Hazard Quotients, HQ) associated with fluoride exposure from food intake in different population groups (25).


(2) $$\begin{array}{l} ADD=\frac{C\;\times \;IR\;\times \;EF\;\times \;ED}{BW\;\times \;AT} \end{array}$$

(3) $$\begin{array}{l} HQ=\frac{ADD}{RfD} \end{array}$$

Where ADD represents the average daily dose (mg·kg^−1^·d^−1^); C is the fluoride concentration in food (mg·kg^−1^); IR is the oral intake amount (kg·d^−1^); EF indicates the exposure frequency, 365 (d·a^−2^); ED is the duration of continuous exposure; AT represents the average exposure time (ED × 365 d·a^−2^); BW is the average body weight; and R*f* D is the reference dose for pollutant exposure (0.06 mg·kg^−1^·d^−1^) (26). When HQ ≥ 1, it suggests potential health risks for the exposed population; when HQ < 1, it indicates no health risk. Oral intake values are based on epidemiological survey data conducted by the research team, with daily food intake calculated from household consumption totals, and children's intake estimated at half that of adults (Table 1) (27). Evaluation parameters are shown in Table 1.

**Table 1:** Values of health risk assessment parameters.

**Type of population**	**Oral Intake Amount (IR) (kg**·**d**^**−1**^**) Median(Min–Max)**				**Average Body Weight(kg)**	**AT (d)**	**EF(d/year)**	**ED (year)**
	**Tofu**	**Corn**	**Pork**	**Chili**				
Children	0.012 (0–0.05)	0.010 (0–0.08)	0.033 (0.004–0.10)	0.028 (0.002–0.08)	25.9	3 285	365	9
Adults	0.023 (0–0.10)	0.021 (0–0.17)	0.067 (0.008–0.20)	0.056 (0.004–0.17)	56.8	25 550	365	70

The cumulative Hazard Index (HI) is evaluated by summing the HQ values generated from the consumption of corn, chili, pork, and tofu, and is calculated as follows (28):


(4) $$\begin{array}{c} HI=HQ_{Corn}+HQ_{Chilli}{+HQ}_{Pork}+HQ_{Tofu} \end{array}$$

When HI > 1, the exposed population may experience potential non-carcinogenic risks; when HI ≤ 1, it is considered that there is no chronic non-carcinogenic risk.

#### 2.3.3 Non-carcinogenic risk probability assessment

There are numerous uncertainties in assessing health risks for populations. To avoid misjudging health risks, this study used Monte Carlo simulation for probability-based risk assessment. Monte Carlo is a mathematical method based on probability and statistics that utilizes random sampling for each parameter to account for uncertainty. By reflecting the probability distributions of variables, it provides a more accurate depiction of real-world conditions (29). In this study, the Monte Carlo method was applied to simulate risk based on fluoride concentration data from corn, chili, pork, and tofu at different roasting stages and population intake levels. Since there were only three values for the food samples at each baking stage, the fluorine content of the different foods was described as a normal distribution by referring to the results of the Shapiro-Elk test during the simulation. The best-fit distributions for the intake of different foods were determined by the Anderson-Darling test, where the optimal distributions for the intake of chili and tofu were the maximum extreme value distributions, for maize the logistic distribution, and for pork the Weibull distribution. Using Monte Carlo simulation, random values were drawn from the fitted distributions for food fluoride content and population intake and input into the U.S. EPA's health assessment model (Equation 3) to calculate the probability distribution of HQ.

### 2.4 Risk threshold

The study hypothesis was based on the population just incurring a health risk (HQ = 1) (30), according to Equations 2, 3. The maximum intake of fluoride-contaminated food by the population (IR_Max_) was derived and shown in Equation 5, which is expressed as the potential health risk that may be caused to occur in the population when the population IR > IR_Max_. The RBA% has also been introduced into IR_Max_ about the bioavailability of fluoride in food to discuss the variation of the maximum permissible daily intake for humans taking into account the function of the gastrointestinal tract, $denoted\;as\;IR_{\text{Max}}^{RBA\%}$.


(5) $$\begin{array}{c} \text{I}{\text{R}}_{\text{Max}}=\frac{\text{HQ }\times \text{ RfD }\times \text{ BW }\times \text{ AT}}{\text{C }\times \text{ EF }\times \text{ ED}} \end{array}$$

### 2.5 Statistical analysis

Data were statistically analyzed using SPSS 27 software, with the Kruskal-Wallis H test employed to analyze differences among experimental groups. A *p*-value of < 0.05 was considered statistically significant. Monte Carlo simulations were conducted in Excel 2016 and Oracle Crystal Ball with 10,000 iterations.

## 3 Results and analysis

### 3.1 Fluoride accumulation in foods roasted with coal

**Figure 1:**
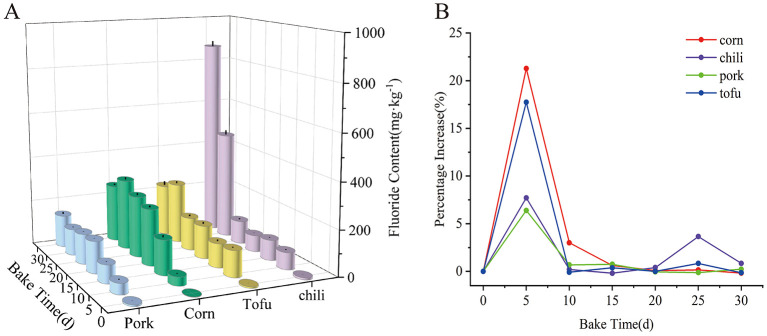
Variation in fluoride content of four foods during roasting. **(a)** Fluorine content. **(b)** Percentage increase in fluorine content with baking time.

The impact of coal roasting on fluoride accumulation in various foods is significant, showing a cumulative effect over time. Initially, fresh pork, corn, tofu, and chili had fluoride concentrations of 6.47, 1.72, 6.19, and 8.56 mg·kg^−1^, respectively, ranking in descending order as: chili > pork > tofu > corn. After roasting with high-fluoride coal briquettes, fluoride levels increased significantly across all food types (*P* < 0.05), reaching peak concentrations of 141.59 mg·kg^−1^ in pork, 308.24 mg·kg^−1^ in corn, 265.70 mg·kg^−1^ in tofu, and 869.82 mg·kg^−1^ in chili (Figure 1A).

The rate of accumulation of fluorine in food increased sharply at the beginning of the roasting period (0–5 days) (in the following order: corn > tofu > chili > pork). After 5 days of roasting, the growth rate of fluoride content started to decrease and then leveled off. In the case of chili peppers, the fluorine content increased slightly and then decreased again after 20–25 days of roasting (Figure 1B). Statistical tests confirmed significant differences in fluoride levels among food types at corresponding roasting stages (*P* < 0.05), with a clear positive correlation between roasting duration and fluoride concentration (*P* < 0.05). These findings emphasize that prolonged roasting with high-fluoride coal substantially raises fluoride levels in foods, with cumulative increases observed across all types.

### 3.2 Bioavailability of fluoride in food

Assessing the bioavailability of fluoride provides critical insights into potential health risks from dietary exposure. For this study, a Caco-2 cell model was employed after intestinal digestive fluid and HBSS buffer was diluted at a 1:4 ratio. Upon meeting the experimental criteria for Caco-2 cell tightness and monolayer integrity, transport assays were conducted. During roasting, chili's bioavailability of fluoride ranged between 2.18 and 12.00%, with a significant increase following roasting (*P* < 0.05); however, the change in bioavailability over time was not substantial. The actual fluoride uptake by chili increased steadily, from an initial 0.19 to 97.97 mg·kg^−1^.

**Figure 2:**
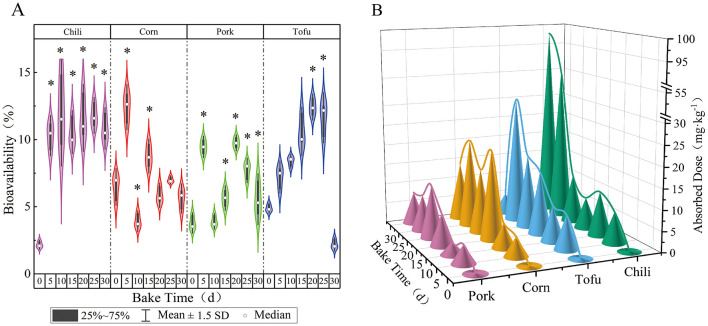
Changes in bioavailability of various foods with roasting time. **(a)** Bioavailability. **(b)** Absorbed dose; *indicates a comparison with the control group, *P* < 0.05.

For corn, fluoride bioavailability fluctuated between 3.92 and 12.42%, with no marked differences before and after roasting (*P* > 0.05). Despite this, the amount of fluoride absorbed by corn from the air increased significantly as roasting progressed, with concentrations rising from 0.11 to 21.71 mg·kg^−1^. Pork exhibited a fluoride bioavailability range of 3.79%−9.76%, showing a minor increase in bioavailability post-roasting (*P* < 0.05); its absorption increased from 0.24 to 12.87 mg·kg^−1^. Tofu's fluoride bioavailability ranged from 2.20 to 11.63%, initially rising during roasting before dropping sharply at 30 days, with absorption increasing from 0.30 to 30.97 mg·kg^−1^. Differences in bioavailability among the four foods were statistically significant at each roasting stage (*P* < 0.05) (Figure 2).

These results further demonstrate a correlation between bioavailability and fluoride accumulation in different foods, suggesting unique absorption characteristics for each food matrix.

### 3.3 Non-carcinogenic risk assessment

#### 3.3.1 Point estimate results of non-carcinogenic risk

**Figure 3:**
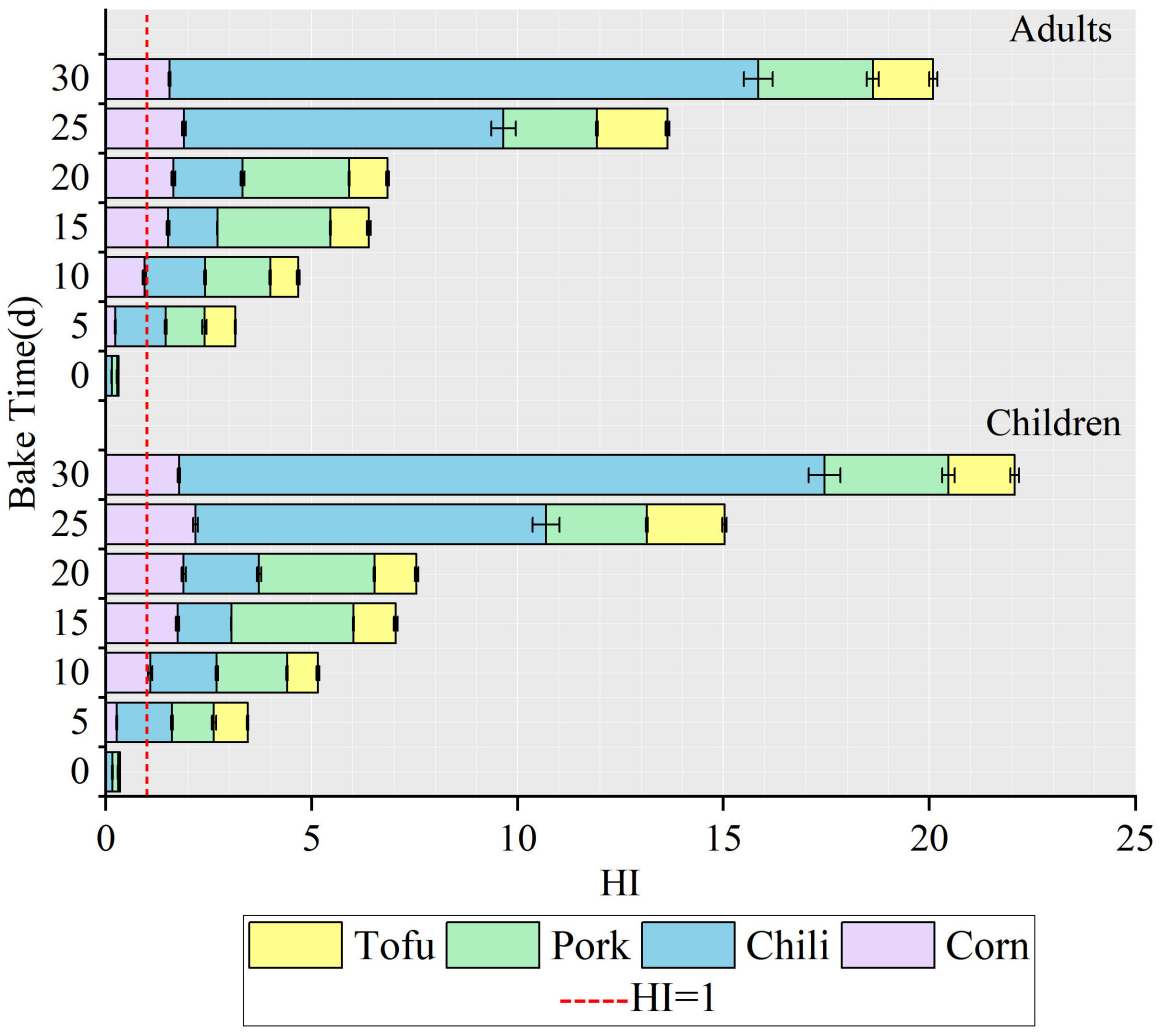
Contribution of four foods to HI between children and adults.

Analyzing the target hazard quotient (HQ) and hazard index (HI) allows a systematic assessment of non-carcinogenic risks associated with dietary fluoride exposure (Figure 3). In the fresh state, HQ values for corn, chili, pork, and tofu were below 1 for both children and adults, with HI values of 0.49 for children and 0.44 for adults, indicating acceptable exposure levels. However, after roasting with high-fluoride coal, HQ values for all foods increased significantly as roasting duration extended (*P* < 0.05). The highest HQ values for children reached 1.98 (corn), 16.79 (chili), 3.10 (pork), and 2.39 (tofu); corresponding HQ values for adults were 1.81, 15.31, 2.78, and 2.10. All exceeded the threshold of 1, indicating potential health risks. The cumulative HI values reached maxima of 47.56 for children and 43.38 for adults, substantially surpassing the acceptable level (HI = 1), signifying a significant risk for individuals consuming these fluoride-laden foods. Data revealed that, regardless of individual HQ or cumulative HI, children face slightly higher health risks than adults, suggesting increased vulnerability to dietary fluoride exposure.

#### 3.3.2 Probabilistic assessment results of non-carcinogenic risk

**Figure 4:**
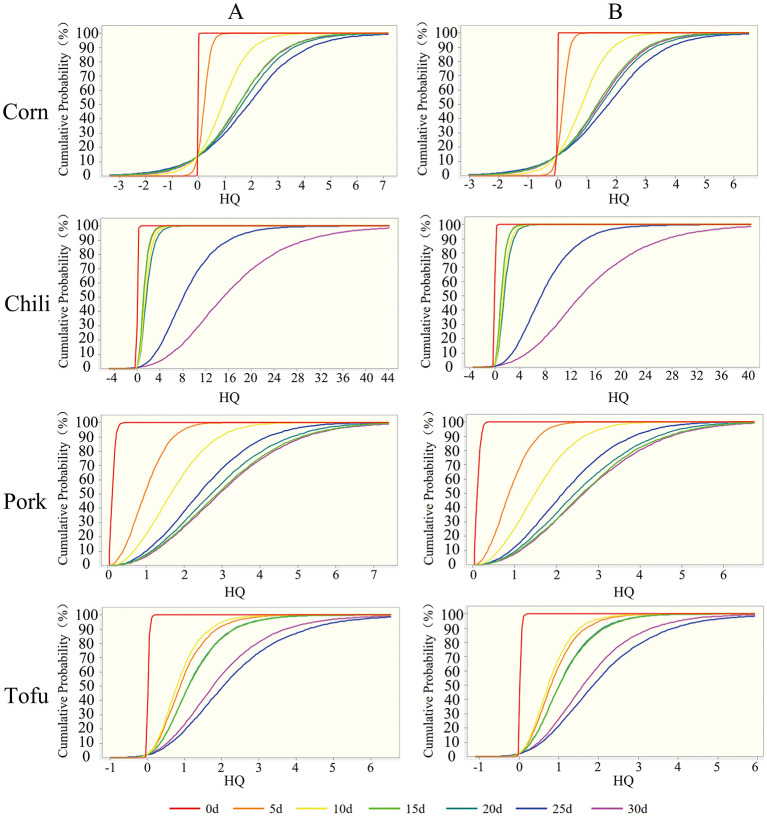
Probability assessment of non-carcinogenic risk from exposure to fluoride-contaminated foods. **(A)** Children; **(B)** Adults.

Using oracle crystal ball for probabilistic risk assessment, the risk evaluation for fluoride-contaminated food was refined (Table 2; Figure 4). Mean HQ and 95th percentile HQ values for fresh corn, chili, pork, and tofu in both children and adults were below 1, indicating low risk. However, after roasting with high-fluoride coal, mean HQ values for corn initially remained below 1 in the first 5–10 days for both groups, while the 95th percentile HQ exceeded safe limits by day 10 (2.51 for children, 2.2 for adults). With extended roasting, mean HQ and 95th percentile HQ values exceeded 1 for all groups, indicating heightened risk.

**Table 2:** Risk assessment results for population exposure to fluoride-contaminated foods based on monte carlo simulation.

	**Bake time**	**Children**			**Adults**		
		**Mean**	**0.05**	**0.95**	**Mean**	**0.05**	**0.95**
Corn	0d	0.01	−0.01	0.03	0.01	−0.01	0.03
	5d	0.24	−0.14	0.62	0.21	−0.13	0.56
	10d	0.93	−0.61	2.51	0.86	−0.50	2.28
	15d	1.53	−0.96	4.06	1.39	−0.87	3.62
	20d	1.65	−1.08	4.30	1.52	−0.93	3.98
	25d	1.91	−1.17	5.02	1.75	−1.02	4.49
	30d	1.56	−0.89	4.06	1.44	−0.83	3.74
Chili	0d	0.16	0.04	0.35	0.13	0.03	0.31
	5d	1.44	0.32	3.04	1.17	0.30	2.70
	10d	1.72	0.40	3.61	1.44	0.37	3.33
	15d	1.40	0.32	2.93	1.14	0.30	2.70
	20d	1.96	0.45	4.15	1.62	0.41	3.71
	25d	9.10	2.09	19.12	7.47	1.91	17.13
	30d	16.80	3.96	35.02	13.73	3.31	32.34
Pork	0d	0.14	0.04	0.27	0.13	0.04	0.25
	5d	1.05	0.30	2.00	0.95	0.28	1.82
	10d	1.77	0.51	3.36	1.60	0.47	3.06
	15d	3.05	0.88	5.84	2.79	0.80	5.31
	20d	2.89	0.85	5.47	2.63	0.77	4.98
	25d	2.54	0.75	4.79	2.30	0.68	4.36
	30d	3.11	0.92	5.90	2.84	0.84	5.42
Tofu	0d	0.05	0.01	0.12	0.05	0.01	0.11
	5d	1.02	0.14	2.29	0.92	0.14	2.04
	10d	0.92	0.14	2.01	0.84	0.12	1.85
	15d	1.28	0.19	2.87	1.16	0.17	2.60
	20d	1.27	0.19	2.83	1.15	0.17	2.53
	25d	2.31	0.34	5.12	2.11	0.30	4.64
	30d	2.01	0.27	4.51	1.82	0.28	4.04

**Figure 5:**
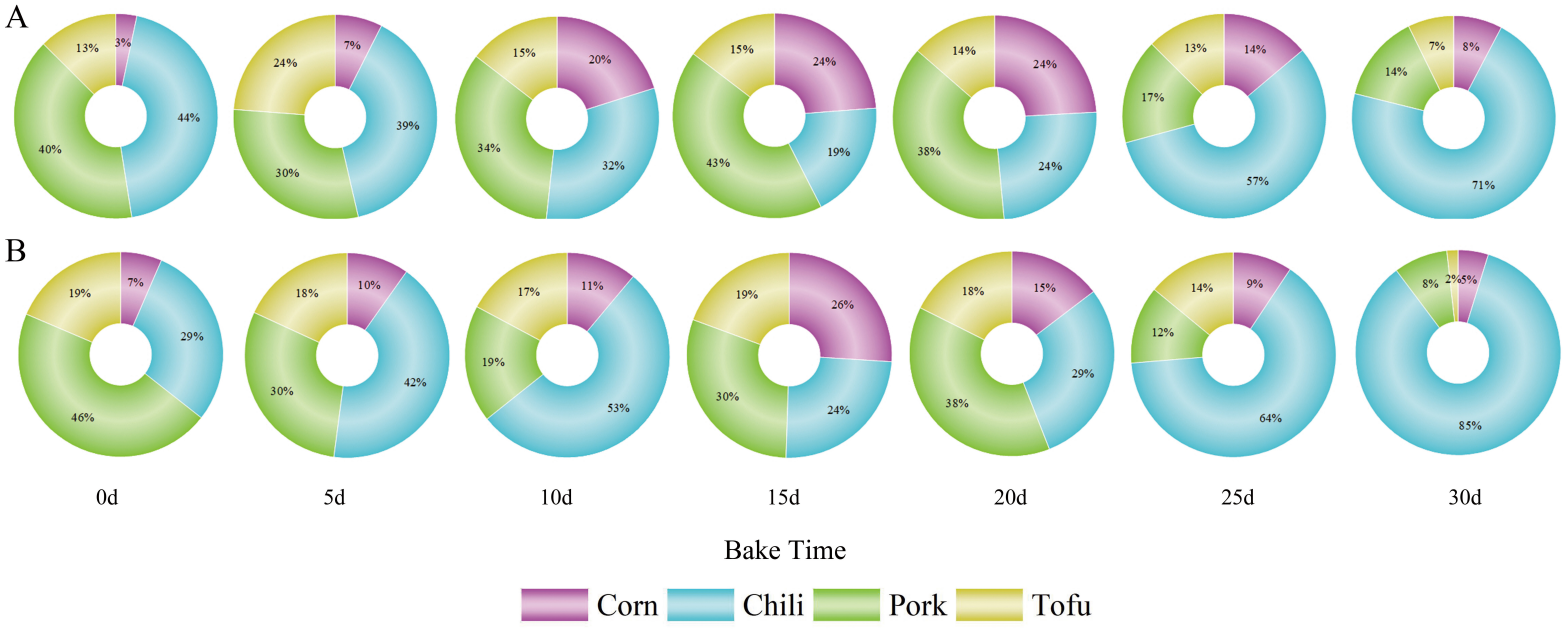
Contribution of four foods to health risk; **(A)** Contribution rate to HI based on total fluoride concentration in each food; **(B)** proportion of absorbed fluoride from each food.

Roasted chili, in particular, displayed HQ values above 1 throughout all roasting stages, with the 5th percentile HQ already exceeding safe levels in the final stages. Similar results were observed for pork, while tofu's HQ exceeded 1 after 10 days, continuing to rise in later stages. These findings underscore that roasted foods pose a substantial health threat as roasting duration increases, with chili representing the most significant contributor to non-carcinogenic risk (Figure 5).

### 3.4 Maximum allowable daily intake

**Figure 6:**
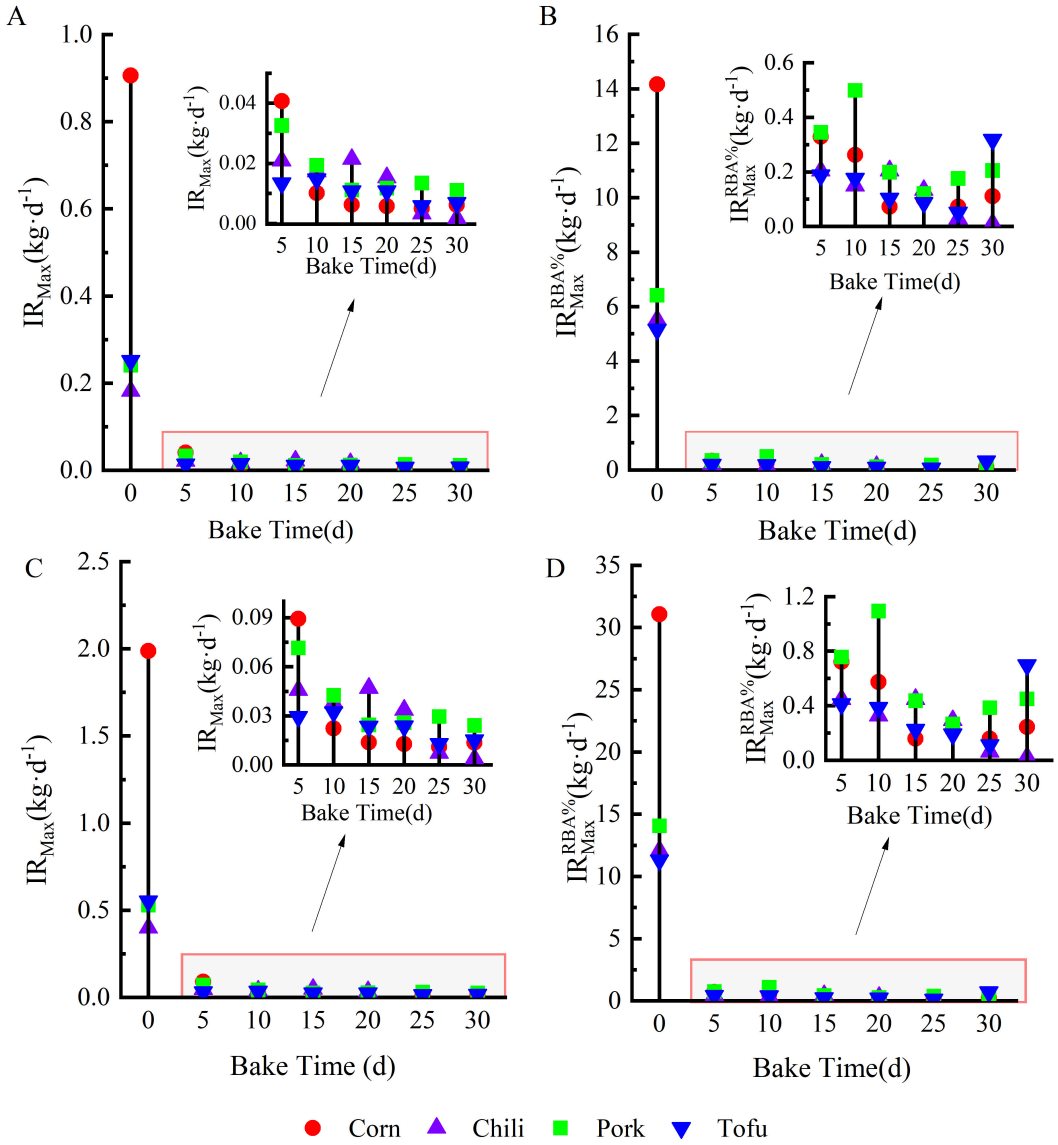
Maximum daily intake (kg·d^−1^) of tofu, pork, chili, and corn. **(A**, **B)** show the maximum daily dose for children based on total dietary fluoride concentration and bioavailable fluoride concentration, respectively. **(C**, **D)** represent the maximum daily dose for adults using the same concentrations, respectively.

The maximum permissible daily intake was calculated based on the health risk threshold (HQ = 1) (Figures 6A, C). For fresh foods, the maximum acceptable daily intake (IR_Max_) was 0.91 kg·d^−1^ (corn), 0.18 kg·d^−1^ (chili), 0.24 kg·d^−1^ (pork), and 0.25 kg·d^−1^ (tofu) for children and 1.99 kg·d^−1^ (corn), 0.40 kg·d^−1^(chili), 0.53 kg·d^−1^ (pork), and 0.55 kg·d^−1^ (tofu) for adults, respectively. Field surveys indicate that residents' actual intake of fresh foods does not exceed these limits, suggesting minimal risk in consuming fresh foods. However, prolonged roasting dramatically reduced the IR values, with children's IR for corn, chili, pork, and tofu decreasing to 0.005, 0.002, 0.011, and 0.006 kg·d^−1^ at peak fluoride accumulation. Adults' IRs similarly decreased to 0.011, 0.004, 0.024, and 0.013 kg·d^−1^, underscoring the potential health risks posed by these foods.

The study calculated corrected maximum daily intake thresholds ($IR_{Max}^{RBA\%}$) by integrating the RBA% of dietary fluoride and showed that ($IR_{Max}^{RBA\%}$) was significantly higher compared to the uncorrected value (IR_Max_) (Figures 6B, D), and that the permissible intake values for corn, pork, and tofu did not exceed the daily intake of children and adults in the field survey at all baking times. It should be noted in particular that at the peak of fluoride accumulation, the ($IR_{Max}^{RBA\%}$) values for chili were only 0.01 and 0.023 kg·d^−1^ for children and adults. Considering the dietary preferences of the local people, especially for roasted chili peppers, chili peppers are likely to be a high prevalence of fluorosis is likely to be a determining factor in these areas.

## 4 Discussion

### 4.1 Fluoride accumulation characteristics and mechanisms in roasted foods

This study confirms that roasting with high-fluoride fuel significantly elevates fluoride accumulation in various foods, and the accumulation patterns differ markedly by food type. Notably, the fluoride concentrations in roasted corn, chili, pork, and tofu observed here exceed levels reported in previous studies. For instance, while Luo and Li noted fluoride accumulation in corn from coal-burning fluorosis areas, their reported concentrations were lower than those in this study (10, 31). Compared to Zeng et al.'s (32) findings on traditional roasting practices, this study observed lower fluoride levels in pork. This underscores the importance of fuel composition in fluoride accumulation assessments, emphasizing that coal source characteristics should be accounted for in regions with coal-burning fluorosis (33).

### 4.2 Sensitivity of different food matrices to fluoride accumulation

This study demonstrates that different food matrices exhibit distinct sensitivities to fluoride accumulation, supporting prior research suggesting that food composition significantly influences fluoride adsorption (14). Corn shows a high potential for fluorine accumulation during the early roasting stage, with the highest increase in fluorine content, which may be related to its food matrix, with the corn germ (especially the corn navel) having an extremely strong adsorption capacity for fluorine (34). Tofu and pork demonstrated high fluoride accumulation potential in the early roasting stages, likely due to their matrix structures, which readily adsorb airborne fluoride (35). The high fluoride enrichment exhibited by chili peppers may be related to their water content characteristics, where fluoride released from coal combustion (mainly in the form of HF and Si_4_) is converted to the acid-soluble form of fluoride and gradually accumulates when it comes into contact with food surface moisture (8, 34). Due to the high water content of chili peppers, it takes longer to achieve complete dehydration under the same baking conditions (19), and prolonged heat exposure facilitates fluoride accumulation. Given the prevalent role of chili in the local diet, its high accumulation of fluoride in coal-fired areas may pose a significant public health risk (5, 11).

### 4.3 Fluoride bioavailability and health risks

This study's application of the Caco-2 cell model to assess fluoride bioavailability provides a more nuanced understanding of dietary fluoride risks, representing a novel approach in coal-burning fluorosis research. While fluoride in drinking water is nearly fully absorbed (28), bioavailability in foods varies significantly (36, 37). The bioavailability of fluorine was significantly higher in chili peppers after roasting, which may be related to the morphological transformation of fluorine during the drying process: dried chili was more likely to release free F^−^ under the digestive action of gastric juices. This was corroborated by data from Li, where water-soluble fluorine accounted for as much as 77.5% of the total fluorine in dried chili peppers, which was much higher than that in fresh chili (9.9%) (38). The reduced bioavailability of corn after roasting may be attributed to a stronger binding of fluorine to the corn matrix (34), suggesting that structural changes in the food matrix during cooking may affect fluorine uptake. Calcium ions abundant in tofu can bind with fluoride to form insoluble salts, such as calcium fluoride, reducing the bioavailability of fluorine in the gastrointestinal tract (39, 40). Therefore, the nutritional community has proposed to reduce the risk of fluorosis through the intake of exogenous calcium supplements (39). These findings indicate that bioavailability, rather than total fluoride content, may provide a more accurate reflection of health risks posed by dietary fluoride in different foods.

### 4.4 Maximum allowable daily intake and health risk control

In regions with prevalent fluorosis, dietary practices such as the consumption of roasted foods contribute to fluoride exposure risks (10, 11, 33, 41). In this study, guideline doses for population consumption of contaminated food were derived based on the concentration of fluorine contamination in food and the bioavailability of fluorine. Children and adults were within the safe intake limits for fresh food under normal consumption patterns. However, extended roasting significantly reduces the IR for these foods due to intensified fluoride accumulation. At peak fluoride accumulation (30), children's IR_Max_ decrease to 0.005 kg·d^−1^ (corn), 0.005 kg·d^−1^ (chili), 0.011 kg·d^−1^ (pork), and 0.006 kg·d^−1^ (tofu), while adult IR_Max_ also decline sharply. This highlights the critical need to control roasted food consumption, especially among children, to mitigate health risks.

Although based on the bioavailability of fluorine can increase the safe threshold of daily intake for both children and adults, the daily intake of children and adults should not exceed 0.01 kg and 0.023 kg for peak accumulation of fluorine in chili peppers, which is a widely consumed condiment in the region, and the maximum daily intake of 0.17 kg for adults, which is already well above the IR_Max_ and $IR_{Max}^{RBA\%}\;$values and is a serious risk factor for fluorosis. Consistent with Yang et al. (11), who noted similar trends with dried chili, this study highlights chili's critical role in dietary fluoride exposure, emphasizing the need to manage high-fluoride chili intake as part of public health strategies.

### 4.5 Health risk assessment

The Monte Carlo probabilistic risk assessment underscores the health risks posed by fluoride in roasted foods, particularly among children. For fresh foods, hazard quotient (HQ), and hazard index (HI) values remain below 1, indicating low risk. However, roasting substantially increases both HQ and HI values, with chili showing the highest HI values for children (47.56) and adults (43.38), far exceeding safe thresholds. This elevated bioavailability in roasted chili underscores its significance in dietary fluoride exposure, especially in later roasting stages, thus emphasizing its potential role in fluorosis development.

Additionally, shifts in dietary habits in high-fluoride regions, including reduced corn intake and increased consumption of chili and pork, exacerbate dietary fluoride risks (5). While corn's decreased intake may reduce its fluoride contribution, increased chili and pork intake could amplify health risks due to its higher bioavailability. These findings suggest that traditional risk assessments focusing solely on total fluoride content may overestimate actual health risks. Integrating bioavailability, as demonstrated in this study, provides a more accurate assessment of dietary fluoride exposure risks and offers insights into effective risk management strategies in fluorosis-prone regions.

## 5 Conclusion

This study systematically evaluated the fluoride accumulation characteristics, bioavailability, and potential health risks of common foods roasted with coal in fluorosis-prone areas of Southwest China, providing essential scientific evidence for dietary fluoride exposure risk management. Results show that chili maintained high fluoride content and bioavailability throughout the roasting process, making it a major risk factor for fluorosis in the population, suggesting that chili may be a critical dietary medium for fluoride exposure in the region. Furthermore, this study identified distinct contributions of corn, tofu, and pork to fluoride exposure risk across different roasting stages. Specifically, corn demonstrated increased fluoride accumulation in the mid-roasting stage, pork showed a gradual rise in bioavailability with extended roasting time, and tofu, despite a decrease in bioavailability after prolonged roasting, continued to contribute to health risks at particular stages. These findings underscore the need to account for multiple dietary sources when assessing population-level fluoride exposure.

By integrating *in vitro* digestion and the Caco-2 cell model, this study accurately identified key dietary sources of fluoride contributing to fluorosis and highlighted the necessity of incorporating bioavailability into risk assessments, providing a more realistic representation of population-level tolerance to various dietary fluoride sources. In conclusion, this study provides scientific support for dietary guidance and risk management in high-fluoride regions of Southwest China, as well as a new perspective for dietary contamination control and public health policy in high-fluoride exposure areas worldwide.

## Data Availability

The raw data supporting the conclusions of this article will be made available by the authors, without undue reservation.
